# Comparative Studies on Two-Dimensional (2D) Rectangular and Hexagonal Molybdenum Dioxide Nanosheets with Different Thickness

**DOI:** 10.1186/s11671-020-03386-x

**Published:** 2020-08-01

**Authors:** Nasrullah Wazir, Chunjie Ding, Xianshuang Wang, Xin Ye, Xie Lingling, Tianqi Lu, Li Wei, Bingsuo Zou, Ruibin Liu

**Affiliations:** 1 Beijing Key Lab of Nanophotonics and Ultrafine Optoelectronic Systems, School of Physics, Beijing Institute of Technology, Beijing, 100081 People’s Republic of China; 2 Guangxi Key Lab of Processing for Nonferrous Metals and Featured Materials and Key lab of new Processing Technology for Nonferrous Metals and Materials, Ministry of Education; Nano and Energy Research Center, School of Physics, Guangxi University, Nanning, 530004 China

**Keywords:** Molybdenum dioxide nanosheets, Hexagonal, Rectangular, Chemical vapor deposition (CVD)

## Abstract

Molybdenum dioxide (MoO_2_) a kind of semi-metal material shows many unique properties, such as high melting point, good thermal stability, large surface area-to-volume ratio, high-density surface unsaturated atoms, and excellent conductivity. There is a strong connection between structural type and optoelectronic properties of 2D nanosheet. Herein, the rectangular and hexagonal types of thin and thick MoO_2_ 2D nanosheets were successfully prepared from MoO_3_ powder using two-zone chemical vapor deposition (CVD) with changing the experimental parameters, and these fabricated nanosheets displayed different colors under bright-field microscope, possess margins and smooth surface. The thickness of the blue hexagonal and rectangular MoO_2_ nanosheets are ~ 25 nm and ~ 30 nm, respectively, while typical thickness of orange-colored nanosheet is around ~ 100 nm. Comparative analysis and investigations were carried out, and mix-crystal phases were indentified in thick MoO_2_ as main matrix through Raman spectroscopy. For the first time, the emission bands obtained in thick MoO_2_ nanosheets via a Cathodoluminescence (CL) system exhibiting special properties of semi-metallic and semi-conductors; however, no CL emission detected in case of thin nanosheets. The electrical properties of thin MoO_2_ nanosheets with different morphologies were compared, and both of them demonstrated varying metallic properties. The resistance of thin rectangular nanosheet was ~ 25 Ω at ± 0.05 V while 64 Ω at ± 0.05 V was reported for hexagonal nanosheet, and observed lesser resistance by rectangular nanosheet than hexagonal nanosheet.

## Introduction

To date, various 2D materials have been synthesized such as graphene, transition metal dichalcogenides, antimony, black phosphorus, Mo_2_C, and h-BN [1–6]; illustrate incredible potential for new type of optoelectronic devices owing to their unique properties and rich in feasibility for the fabrication of 2D materials technologies [7]. Certainly, some of specific 2D materials have shortcomings such as zero band gap, low absorption efficiency, and instability in open atmosphere are the challenges in fabrication of ideal nanoscale devices. In order to overcome these challenges, the transition metal oxides (TMOs) have been found effective 2D materials in terms of possessing high conductivity, piezoelectricity, colossal magnetoresistance, better stability in open environment and superconductivity, etc. [8–10]. Molybdenum dioxide is a typical TMOs material having three crystalline polymorphic forms; hexagonal phase (P6_3_/*mmc*) [11], tetragonal phase (P4_2_/*mnm*) [12] and monoclinic (P2_1_/c) [13], and also possesses a partially rutile configuration [14] containing MoO_6_, octahedrally linked Mo through oxygen atoms in the edges of unit cell involves four MoO_2_ units opposite to two unit cells [15, 16]. It is well known that the properties of molybdenum oxides are strongly dependent on their crystalline structures; in particular, the rutile structure of MoO_2_ is interesting due to possession of superb metallic-like electrical conductivity [12], low electrical resistivity, high melting point [17, 18], facile ion transport [19], and excellent chemical stability [20]. It has been interrelated to various interatomic bondings and comparatively over the top density of states at the Fermi level. The presence of free electrons generate Mo^4+^ in MoO_2_ in contrast to generation of Mo^6+^ from MoO_3_; hence, all the valence electrons in molybdenum metal are covalently bonded to nearest oxygen atoms [21, 22]. A small variation in Mo valence may cause significant fluctuations in physical properties of the molybdenum oxides. For instance, it is possible to obtain compounds of other oxides with diverse physical properties [23, 24].

Therefore, the crystallinity, shape, and size of product can be achieved smoothly by changing various parameters under desired synthesis techniques; for example, Spevack et al. obtained monoclinic structure MoO_2_ (P2_1_/c) from ɑ-MoO_3_ by thermal reduction method [25]. Alves et al. reported the electronic and structural transitions at various temperature and resulted in increasing thermal expansion, heat capacity, and electrical resistivity of single-crystal MoO_2_ [26]. Jacob et al. described the deformation in MoO_2_ at high temperature and acknowledged a phase transition occurring at specific temperature with transformation of distorted rutile structure (*P*2_1_/*c*) into a hexagonal rutile structure (*P*4_2_/*mnm*) [27]. In addition, the electronic structure and properties of molybdenum oxide materials vary with thickness [16], and MoO_2_ nanostructures have been widely used in electro-chemical supercapacitors [28], catalysis [18], sensing [29], energy storage [30], electrochromic displays [31], and energy conversion regimes [32] because of their superior charge transport properties [24]. Furthermore, various methods have been used for synthesis of diverse morphologies MoO_2_ for achievement of exceptional properties. MoO_2_ has no van der Waals crystalline property and hence, cannot be exfoliated to layers from bulk. Mostly, MoO_2_ has been synthesized from their precursors by different techniques such that hydrothermal and solvothermal routes [11], thermal decomposition of molybdates [33], solid reduction reaction [34], and electrospinning [35] having varied morphologies, such as nanoparticles [36], nanowires [31], nanorods [28], nanostars [28], nanosheets [37], hollow [38], and mesoporous particles [39]. However, these methodologies are found ineffective to control the surface morphology and size of particles [22].

Two-dimensional MoO_2_ nanosheets with thin and well homogenous surface morphology have been considered suitable for high metallic conductivity, perfect chemical stability, and enable 2D MoO_2_ nanosheets as promising for integration of 2D materials in a variety of electronic structures and nanoscale devices [40]. Here, we have presented a comparative study and synthesis of two-dimensional (2D) rectangular and hexagonal molybdenum oxide nanosheets supported on SiO_2_/Si without post-annealing treatment via CVD technique: the two types of ultrathin MoO_2_ nanosheets with various thickness were successfully prepared and characterized by Raman, AFM, and CL, as well as I–V characterizations. The electrical behavior of the molybdenum oxides varies from semi-metal to wide bandgap semi-conductor as it depends on thickness and oxides state. The time adjustment controls the deposition, thickness, and determines the sub-oxide states [41]. Insight into the oxides’ phase stability, ranges, and mixtures are not only significant for understanding molybdenum oxide nanosheets, but also important for other TMOs for optoelectronic applications [42].

## Methods/Experimental Section

### Synthesis of the Hexagonal MoO_2_ Nanosheets

**Fig. 1:**
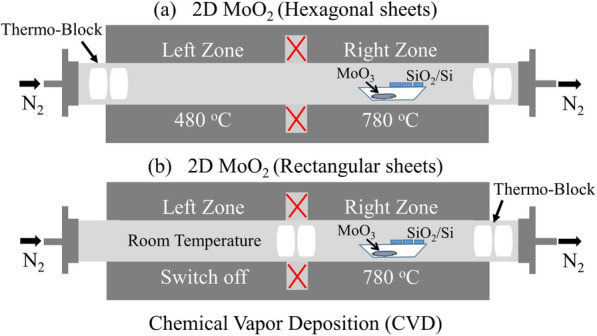
Schematic configuration of the CVD system (the red cross represents insulating regions). **a** Hexagonal molybdenum oxides. **b** Rectangular molybdenum oxides

Synthesis of hexagonal molybdenum dioxides (MoO_2_) nanosheets from precursor ~ 20 mg of MoO_3_ powder (99.95%, Alfa Aesar) placed at one end of quartz tube in a porcelain boat and heated in two-zone furnace under nitrogen (N_2_) atmosphere as shown in the Fig. 1a. The parent SiO_2_/Si substrates are sequentially cleaned with deionized water, acetone, ethanol, and isopropanol through sonication and arranged the clean substrates at 3 cm distance from precursor powders. Two thermal blocks were placed at the end of the quartz tube; before heating, the quartz tube was purged with a N_2_ (99.999%) at a constant gas flow rate of 200 sccm for 20 min to remove O_2_ and other contaminants and then reduced flow rate to 20 sccm as a carrier gas. The left heating zone was set up to 480 °C at rate of 10 °C min^−1^ rise in temperature, while the right zone was set up to 780 °C at the same rate of rising temperature, and held for 20 min in the presence of N_2_ environment. After the completion of reaction, the furnace was left to naturally cool down to room temperature and finally obtain hexagonal MoO_2_ nanosheets deposited on the SiO_2_/Si substrates.

### Synthesis of the Rectangle MoO_2_ Nanosheets

In similar fashion mentioned above, the rectangle molybdenum dioxide (MoO_2_) nanosheets were grown in two-zone tube furnace under N_2_ atmosphere, as shown in the Fig. 1b. In this set up, the thermo-blocks were placed close to the middle of the tube: left zone was set at room temperature and the rest of the parameters were remained the same, as were set for the synthesis of hexagonal MoO_2_ nanosheets, to grow rectangular MoO_2_ nanosheets on the SiO_2_/Si substrates.

### Fabrication of the Devices

E-beam lithography was followed for fabrication of electrodes with Ti (10 nm)/Au (90 nm) for hexagonal and the rectangular MoO_2_ nanosheets as contact.

### Characterizations

AFM images were obtained using atomic force microscope, a Dimension Edge PSS (Bruker, Inc., Karlsruhe, Germany) in a non-contact mode. SEM images were recorded under a Hitachi S-4800 microscope working at 10.0 kV. Optical photographs were recorded by an optical microscope (Olympus BX51M). Raman spectra was acquired by a confocal Raman setup (based on spectrometer Princeton Acton SP2500). Cathodoluminescence (CL) spectra was captured by a CL measurement setup (Horiba Is-100-em-type2). I–V curves of the devices were measured by a micro probe station system (Keithley 4200-SCS).

## Results and Discussions

Different conditions were set for synthesis of rectangular and hexagonal TMO’s nanosheets, and the changing parameters resulted in different shaped nanosheets as shown in Fig. 1. The literature suggests some possible reaction mechanism for CVD-grown nanosheets; some of nitrogen gas molecules are converted into ionized nitrogen molecules at specific temperature by thermal irradiation in a tube furnace, and such ionized nitrogen molecules are marked by ${\mathrm{N}}_2^{\ast }$ [43]. At the desired temperature, the molecules of MoO_3_ collide with the ionized ${\mathrm{N}}_2^{\ast }$ molecules, which produce a series of possible reactions in the presence of inert nitrogen gas environment [44–46].

1 $$ \mathrm{e}+{\mathrm{N}}_2\to {\mathrm{N}}_2^{\ast }+\mathrm{e} $$

2 $$ {\mathrm{M}}_{\mathrm{o}}{\mathrm{O}}_3+\left(\frac{x}{2}\right){\mathrm{N}}_2^{\ast}\to {\mathrm{M}}_{\mathrm{o}}{\mathrm{O}}_{3-\mathrm{x}}+\mathrm{xNO} $$

3 $$ {\mathrm{M}}_{\mathrm{o}}{\mathrm{O}}_{3-\mathrm{x}}+\left(\frac{1-x}{2}\right){\mathrm{N}}_2^{\ast}\to {\mathrm{M}}_{\mathrm{o}}{\mathrm{O}}_2+\left(1-\mathrm{x}\right)\ \mathrm{NO} $$

4 $$ {\mathrm{M}}_{\mathrm{o}}{\mathrm{O}}_2+3{\mathrm{M}}_{\mathrm{o}}{\mathrm{O}}_3\kern0.5em \to {\mathrm{M}}_{\mathrm{o}4}{\mathrm{O}}_{11} $$

The structure of evaporated MoO_3_ molecules may change to different kinds of morphologies nanosheets, either by increasing or decreasing N_2_ gas flow and the holding time at desired temperatures [45]. During the diffuse of molecules toward substrates, they start aggregation to form different kinds of regular rectangular and hexagonal MoO_2_ sheets.

**Fig. 2:**
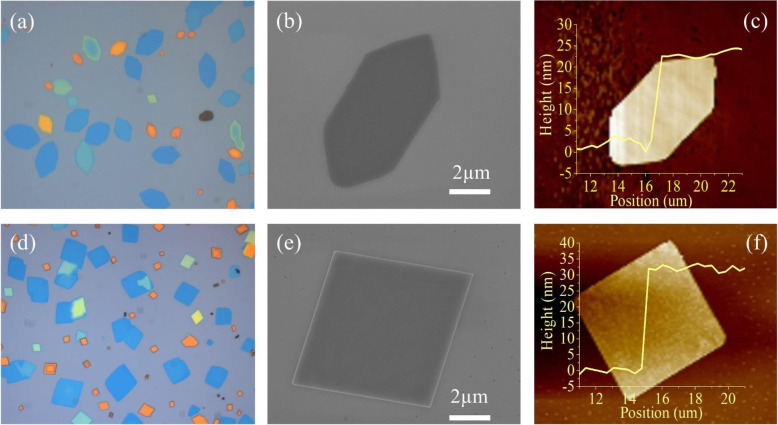
**a** Photograph of hexagonal molybdenum oxide nanosheets under microscope. **b** SEM image of hexagonal molybdenum oxide nanosheet, Scale bar 2 μm. **c** AFM result of blue-colored hexagonal molybdenum oxide nanosheet. **d** Photograph of rectangular molybdenum oxide nanosheets under the microscope. **e** SEM image of rectangular molybdenum oxide nanosheet on scale bar 2 μm. **f** AFM result of blue-colored rectangular molybdenum oxide nanosheet

Figure 2a shows CVD fabricated hexagonal MoO_2_ nanosheets far better than the solution-based synthesis of hexagonal MoO_2_ nanosheets [47, 48]. Furthermore, different phase structures of rectangular MoO_2_ can be obtained using CVD technique with certain control parameters; the temperature of heating zones, location of thermal blocks, and position of substrates as shown in Fig. 2d. Fixing the temperature of left heating zone at 480 ^°^C was extremely important for synthesis of hexagonal MoO_2_ nanosheets. The basic mechanism of hexagonal MoO_2_ nanosheets is a temperature gradient. Xu, X., et al. report high dependency of morphologic variation is thermodynamics and kinetics influence in crystal growth process, which is based on the difference in thermodynamic stability and the lattice strain between the phases [49]. Both low- and high-temperature zones play crucial role in fabrication of hexagonal nanosheets; however, the variation in temperature of high-temperature zone is very effective for growth of rectangular MoO_2_ nanosheet. In addition, Wang, S., et al. reported an evolution of two-dimensional nanosheets that are highly dependent upon the spatial location of the substrates [50]. Yang, X., et al. also reported the temperature-dependent growth of regular nanosheets morphology and further explained the chemical vapor mechanism with the help of first principle KMC method [51]. There are some blue- and orange-colored hexagonal and rectangular nanosheets of different thickness; blue-colored nanosheets are thinner than orange-colored nanosheets and are in close agreement with characteristics of other 2D materials [52]. Mixed colors mean a layered nanosheet formed various thicknesses, as shown in Fig. S1 and Fig. S2 in supplementary information. The thickness of the nanosheets monotonically decreased in sequence: orange color, yellow color, and blue color, which depends on the variation of synthetic parameters. Figure 2b, e shows the amplified SEM image of the typical hexagonal and rectangular nanosheet, illustrating smooth surface, clear margins, regular shape of high quality, and 10 μm in length. The AFM measured the blue hexagonal and rectangular nanosheets as ~ 25 nm and ~ 30 nm thick, respectively, as shown in the Fig. 2c, f.

**Fig. 3:**
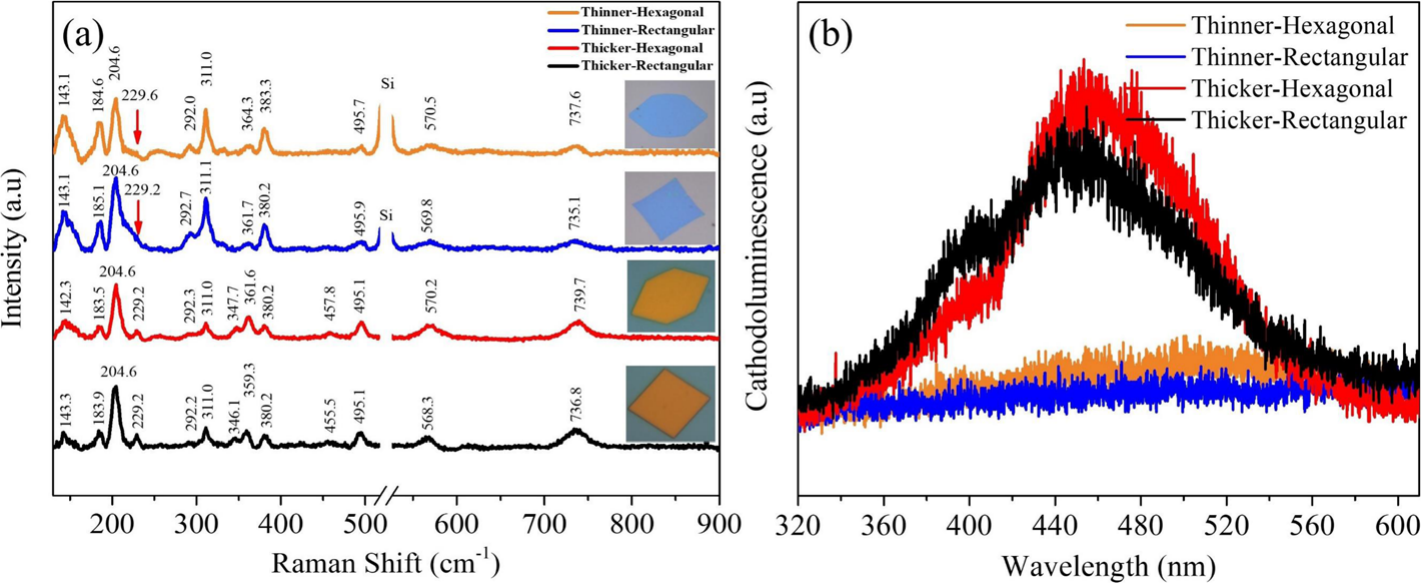
**a** Raman spectra of blue-colored thin hexagonal and rectangular molybdenum oxide nanosheets and orange-colored thick hexagonal and rectangular molybdenum oxide nanosheets. **b** Cathodoluminescence (CL) spectra (orange spectra for thin hexagonal nanosheet, blue spectra represent thin rectangular nanosheet, red spectra represent thick hexagonal nanosheet, and black spectra represent thick rectangular nanosheet)

Raman spectra was acquired to investigate the quality and uniformity of as-grown MoO_2_ nanosheets. Here, we present Raman spectra of rectangular and hexagonal MoO_2_ nanosheets having different colors under the irradiation of 532 nm laser. In Fig. 3a, the Raman peaks obtained from thin hexagonal nanosheet (blue color) locate at 143.1, 184.6, 204.6, 229.6, 292.0, 311.0, 364.3, 383.3, 495.7, 570.5, and 737.6 cm^−1^, respectively. In comparison to the thin rectangular nanosheet, blue color peaks are consistent with a little peak shift at 143.1, 185.1, 204.6, 229.2, 292.7, 311.1, 361.7, 380.2, 495.9, 569.8, and 735.1 cm^−1^, respectively. Both types of thin blue-colored nanosheets got the same numbers of Raman peaks; however, an additional strong silicon’s peak was obtained at 526 cm^−1^ in blue-colored hexagonal nanosheet. In fact, the peak shift originates from the difference in thickness of nanosheets; hexagonal nanosheets are thinner than rectangular nanosheets, as shown in Fig. 2c, f. The additional peak location for silicon happened from laser penetration, hitting silicon surface, served as substrate, and attributed to blue-colored hexagonal and rectangular nanosheets’ thickness compared to orange-colored thick hexagonal and rectangular nanosheets, as shown in Fig. 3a. For thick hexagonal nanosheets, 13 peaks were obtained with the peak position at 142.3, 183.5, 204.6, 229.2, 292.3, 311.0, 347.7, 361.6, 380.2, 457.8, 495.1, 570.2, and 739.7 cm^−1^, respectively. The 13 peaks noted for thick rectangular nanosheet at position of 143.3, 183.9, 204.6, 229.2, 292.2, 311.0, 346.1, 359.3, 380.2, 455.5, 495.1, 568.3, and 736.8 cm^−1^ having small variation in position compared to hexagonal nanosheets. The thickness induced few additional peaks at different wavenumber as compared to the blue-colored thinner nanosheets [53]. The identification details of Raman peaks for thin/thick hexagonal and rectangular nanosheets are given in Fig. S3 in supplementary file; the results are well agreed with the reported results of monoclinic MoO_2_ thin film fabricated by different CVD synthesis routes [54, 55]: thickness and peaks shifting depend on growth’s conditions [56, 57]. In the present work, for the first time, we report 13 vibrational peaks for orange color, while 11 peaks for blue-colored regular hexagonal and rectangular MoO_2_ nanosheets, confirming the existence of mixed structures in MoO_2_ nanosheets. The sharp and strong peaks confirmed better crystallinity compared to other reported results [15, 54, 55].

We can conclude from these four typical nanosheets that all of them contain a complex and mix structures, such as pure MoO_3_, MoO_3−x_, monoclinic MoO_2_, orthorhombic MoO_3_ (α-MoO_3_), and orthorhombic Mo_4_O_11_. The Raman peak at 289 cm^−1^ is assigned to pure MoO_3_, 142 cm^−1^ to MoO_3−x_ [58] and the peak at 287 cm^−1^ is associated with orthorhombic α-MoO_3_ [59]. Dieterle, M. reported Raman peaks for different molybdenum oxides; orthorhombic MoO_3_, monoclinic MoO_2_, and orthorhombic Mo_4_O_11_; the bands at 290–292 cm^−1^ are considered to be raised from orthorhombic MoO_3_ (α-MoO_3_), while Raman peaks at 183, 306 cm^−1^ raised from orthorhombic Mo_4_O_11_ [60]. The peaks at 380 cm^−1^ were assigned to MoO_2_ [61], and 460 cm^−1^ to α-MoO_3_ [62]. The Raman spectra results of our synthesized nanosheets are presented in Fig. 3a. Raman spectra results of individual nanosheets are available in supplementary Fig. S3. Therefore, the Raman peaks in our results are associated with different structural phases of various molybdenum oxides: 142.3 ~ 143.3 cm^−1^ (MoO_3−x_), 183.5 ~ 185.1 cm^−1^ (Mo_4_O_11_), and 204.6 cm^−1^ (MoO_2_). Moreover, peaks at 229.2 ~ 229.6 cm^−1^ (MoO_2_) present in orange-colored nanosheets are sharper and broader compared to blue-colored nanosheets, which confirm existence of multiple strains in thick nanosheets. The peaks at 292.0 ~ 292.7 cm^−1^ (α-MoO_3_) in blue-colored nanosheets are sharper and broader than orange-colored nanosheets. The peaks at 311.0 ~ 311.1 cm^−1^ (Mo_4_O_11_) exist in all four types of nanosheets; the more intense peak is in thin blue-colored nanosheets compare to orange-colored nanosheets. However, the peaks at 346.1 ~ 347.7 cm^−1^ (MoO_2_) only exist in orange-colored nanosheets. Mostly, all these peaks are present in all kinds of nanosheets with little variation; peaks at 359.3 ~ 364.3 cm^−1^ for (MoO_2_) and peaks at 380.2 ~ 383.3 cm^−1^ (MoO_2_) were present in all nanosheets; however, the peaks at 455.5 ~ 457.8 cm^−1^ (α-MoO_3_) are only present in orange-colored nanosheets. Major peaks are well matched with molybdenum dioxides and raised in all nanosheets, e.g., peaks at 495.1 ~ 495.9 cm^−1^ (MoO_2_), 568.3 ~ 570.5 cm^−1^ (MoO_2_) and 735.1 ~ 739.7 cm^−1^ (MoO_2_). The sub-oxides happened due to inter valence transitions; in sub-oxides, the distance between Mo atoms are associated with oxygen atoms, in which laterally tetrahedral *c* axis increased with increasing distortion of the bond from undisturbed portion to shear plane. This affects the completely polarized modes parallel to *c* axis: the polarized modes perpendicular to *c* axis are affected by the distances of M=O bond. The escaping of oxygen atoms from pure MoO_3_ after treatment with high temperature confirmed shear crystallographic structures via prolonged shear defects, finishing the translational symmetry [58]. The larger numbers of extra peaks are noted for orange-colored hexagonal and rectangular nanosheets other than the regular peaks of molybdenum dioxides; the peaks at 142.3 ~ 143.3 cm^−1^ for MoO_3−x_, the peaks at 292.2 ~ 292.3, and 455.5 ~ 457.8 cm^−1^ for α-MoO_3_, and peaks at 183.5 ~ 183.9 and ~ 311.0 cm^−1^ for Mo_4_O_11_.

For further verification, the Cathodoluminescence (CL) is carried out to verify the structural phase’s impact of complex molybdenum oxide structural phases on the metallic properties of both thick hexagonal and rectangular nanosheets. Theoretically, the semi-metallic MoO_2_ will partially transform into semi-conductor due to involvement of MoO_3_. However, it is very difficult to measure the PL spectrum of pure MoO_2_ nanostructures due to the metallic characteristics: MoO_3_ is a wide bandgap semi-conductor with a weak luminescent intensity until conversion and disappearance of MoO_3_ to MoO_3−x_, and further conversion to MoO_2_ nanosheets [63]. Hence, no luminescent spectrum reported for hexagonal and rectangular molybdenum oxide nanosheets.

The Cathodoluminescence (CL) properties of molybdenum thick nanosheets revealed the electronic transition between the conduction and valence bands due to the presence of suboxides. As shown in the Fig. 3b, the CL spectra acquired for thick hexagonal and rectangular molybdenum oxide nanosheets show a small CL peaks at 410 nm (3.02 eV), while a stronger and wider peaks at 454 nm (2.73 eV) generated from nanosheets. The weaker peaks at 410 nm in both CL spectra are similar to MoO_3_ spectra, and the weak emission may be associated with a trap state recombination: 454 nm band assigned to defect-related trap-states originated from oxygen vacancies [64]. The emitted photon energy for pure MoO_3_ located at 3.02 eV instead of 3.2 eV for bandgap indicates the conversion of MoO_3_ powder into MoO_3−x_ and was verified via Raman spectra results. The broadband CL spectra that vary from 3.02 to 2.73 eV confirmed the mixed nanostructures in thick nanosheets, possessing metal-semi-conductor gradient behaviors. These molybdenum oxides are related to the development of carrier concentrations, oxygen vacancy concentrations in reduced MoO_3_, and free-electron concentration in MoO_2_. These studies advanced the knowledge of structural and optical properties of sub-stoichiometric molybdenum oxide nanosheets, contributing to the development of advanced optical devices. Similar CL spectra are reported for other transition metal oxides, such as WO_3_ and α-Fe_2_O_3_ [65]. The CL spectra detection issue in thin nanosheets is described and reported in literature. The CL intensity of the flakes decreased with the decrease in layer thickness; thin-layered flakes are transparent to electron beam; created electron-holes are directly proportional to their thickness [66]. Bourrellier, R., et al. verified broadband luminescence detection in low-quality crystals, which is not related with extrinsic defects, but obviously with intrinsic defects that can be generated by electron irradiation [67]. Recently, Zhou, N., et al. reported strong CL intensity orientation from increasing of defect concentration and increasing flake thickness, but the intensity of CL emission decreased with the decrease of flake thickness [68]. In spite of this effect, the thick flakes show a significant luminescence in comparison to the thin flakes. This is the reason that CL spectra of pure thin MoO_2_ cannot be detected in thin nanosheets; the CL spectra of both thin hexagonal (orange-colored spectra) and rectangular nanosheet (blue-colored spectra) are presented in Fig. 3b.

**Fig. 4:**
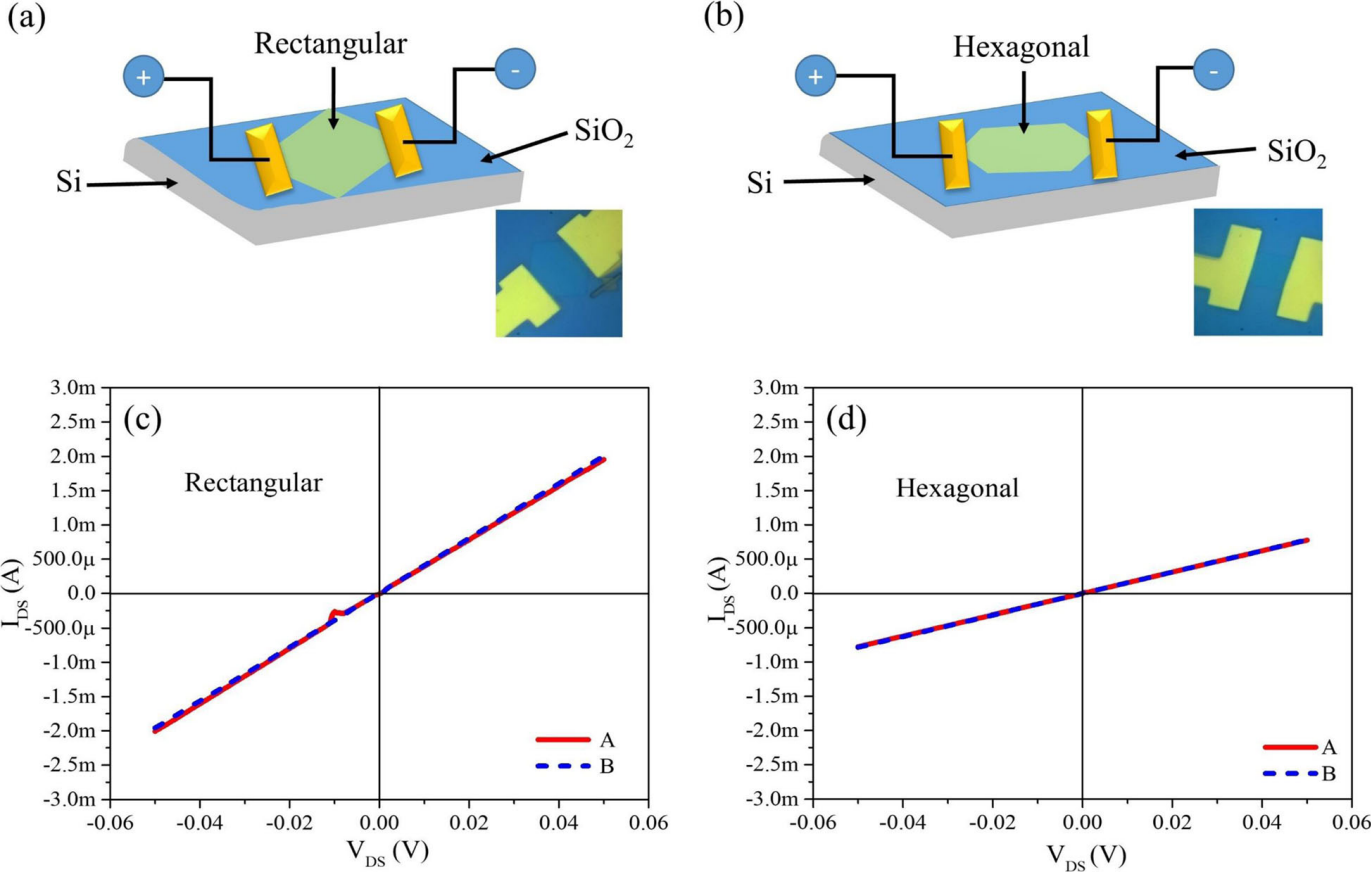
**a** Schematic diagram of single rectangular molybdenum oxide nanosheet’s device. Inset: real image of the device. **b** Schematic diagram of single hexagonal molybdenum oxide nanosheet’s device. Inset: real image of the device. **c** I–V characteristics of individual single rectangular molybdenum oxide nanosheet’s device. **d** I–V characteristics of individual single hexagonal molybdenum oxide nanosheet’s device

The thin hexagonal and rectangular nanosheets possess metallic characteristics confirmed by I–V measurement, as shown in Fig. 4 and supporting information (SI) in Fig. S4. We have fabricated two terminals containing six devices on blue-colored thin nanosheets and measured I–V curves with same parameters; three of them were rectangular nanosheets, and the rest of the three were hexagonal nanosheets, as shown in the inset of Fig. 4a, b, respectively. The contacts were fabricated with Ti/Au as electrodes. The schematic diagram of single rectangular and hexagonal molybdenum oxide nanosheets’ devices are shown in Fig. 4a, b. In Fig. 4c, d, the I–V curves of both types of nanosheets are measured by sweeping the bias voltage from negative (− 0.05 V) to positive (+ 0.05 V) for several times without show any variation in devices and displayed linear behavior with Ohmic contact between the nanosheets and electrodes of devices. Ohmic equation was utilized for resistance measurement, *R* = *V*/*I*, where *R* represents resistance, *V* voltage, and *I* current; resistance of rectangular and hexagonal nanosheets were measured ~ 25 Ω at ± 0.05 V and 64 Ω at ± 0.05 V, respectively, which further confirmed that rectangular nanosheet has less resistance than hexagonal nanosheet.

Additionally, the resistance (*R*) of blue-colored thin rectangular nanosheets are ~ 30 Ω at ± 0.05 V and ~ 43 Ω at ± 0.05 V as shown in Fig. S4a, b in supporting information (SI); however, resistance (*R*) of blue-colored thin hexagonal nanosheets are ~ 61 Ω at ± 0.05 V and ~ 61 Ω at ± 0.05 V as shown in Fig. S4c, d. This verifies that the blue-colored thin rectangular nanosheets have lesser resistance at the same parameters than blue-colored thin hexagonal nanosheets.

## Conclusions

In this work, we report the control-synthesis of rectangular and hexagonal molybdenum oxide nanosheets from single precursor powdered MoO_3_ without post-annealing treatment through CVD methods. Comparative analysis and investigations were carried out using different spectroscopic techniques: Raman spectra, optical photograph, scanning electron microscopy, atomic force microscopy, and Cathodoluminescence (CL). The optical contrast depends on the thickness of nanosheets. SEM results confirmed well-symmetry and smooth morphology of controlled nanosheets. AFM analysis measured ~ 30 nm thickness of thin rectangular nanosheets, and ~ 25 nm hexagonal nanosheet. Raman spectra results reveal existence of mixed structures in MoO_2_ nanosheets due to the complex crystalline structures. Strong spectral response and peaks shifting depends on thickness of nanosheets. Comparatively less Raman peaks were observed for thin than thicker nanosheeet, and well matched with the vibrations of crystal MoO_2_ and other mixed crystals; however, some peaks disappeared in thin 2D nanosheets. The thick orange-colored nanosheets contain more peaks due to the complex structural phases of molybdenum oxide; especially, additional MoO_3_ and MoO_3−x_ occurs in semi-metallic MoO_2_, and thus the thick nanosheets exhibit wide bandgap semi-conductor behaviors and were further verified by Cathodoluminescence (CL) spectra. For the first time, a combined metallic and wide bandgap semi-conductor properties were observed in mixed molybdenum oxide, thick hexagonal and rectangular nanosheets. These peaks in orange-colored nanosheets might be helpful photonic materials for practical applications in nanoscale devices; however, no CL emission is detected for thin nanosheets. The I–V curves of all devices fabricated thin rectangular or hexagonal nanosheets demonstrated linear metallic behavior due to well-established Ohmic contact between nanosheets and electrodes. The thin hexagonal nanosheet exhibited higher resistance than rectangular nanosheet. This study provides a deep comprehension of special 2D molybdenum oxide nanosheets, providing a way for modulating the properties of different types of nanosheet.

## Additional Files

**Additional file 1: Figure S1*.*** The optical images reveal difference between the thickness of nanosheets associated with different colors. **Figure S2.** Optical microscopic images of nanosheets having different sizes and colors. **Figure S3.** Raman spectra of individual hexagonal and rectangular molybdenum dioxide nanosheets. **Figure S4.** The I-V curves of both blue color thin rectangular and hexagonal nanosheets devices.

## Data Availability

All data are fully available without restriction.

## Abbreviations

2D: Two dimensional
MoO_2_: Molybdenum dioxide
MoO_3_: Molybdenum trioxide
CL: Cathodoluminescence
R: Resistance
Ω: Ohm
V: Voltage
I–V: Current–Voltage
CVD: Chemical vapor deposition
Mo_2_C: Molybdenum carbide
h-BN: hexagonal-Boron nitride
TMOs: Transition metal oxides
P: Space group
N_2_: Nitrogen
$$ {\mathrm{N}}_2^{\ast } $$: Ionized nitrogen
sccm: Standard cubic centimeters per minute
SiO_2_/Si: Silicon dioxide/silicon
Ti: Titanium
Au: Gold
AFM: Atomic force microscope
SEM: Scanning electron microscope
