# Supplementary material for: Comparative Studies on Two-Dimensional (2D) Rectangular and Hexagonal Molybdenum Dioxide Nanosheets with Different Thickness
Source: Nanoscale Res Lett. 2020 Aug 1;15:156. doi: 10.1186/s11671-020-03386-x (PMC7395921; doi:10.1186/s11671-020-03386-x)
Supplement: Supplementary file 1 — Additional file 1: Figure S1. The optical images reveal difference between the thickness of nanosheets associated with different colors. Figure S2. Optical microscopic images of nanosheets having different sizes and colors. Figure S3. Raman spectra of individual hexagonal and rectangular molybdenum dioxide nanosheets. Figure S4. The I-V curves of both blue color thin rectangular and hexagonal nanosheets devices. [file 11671_2020_3386_MOESM1_ESM.docx]

**Supplementary information**

**Comparative Studies on Two-Dimensional (2D) Rectangular and Hexagonal Molybdenum Dioxide Nanosheets with Different Thickness**

*Nasrullah Wazir, Chunjie Ding, Xianshuang Wang, Xin Ye, Xie Lingling, Tianqi Lu, Li Wei, Bingsuo Zou*, Ruibin Liu**

*Beijing Key Lab of Nanophotonics and Ultrafine Optoelectronic Systems, School of Physics, Beijing Institute of Technology, Beijing, 100081, P. R. China*

*E-mail: [liuruibin8@gmail.com](mailto:liuruibin8@gmail.com); [Zoubs@bit.edu.cn](mailto:Zoubs@bit.edu.cn)


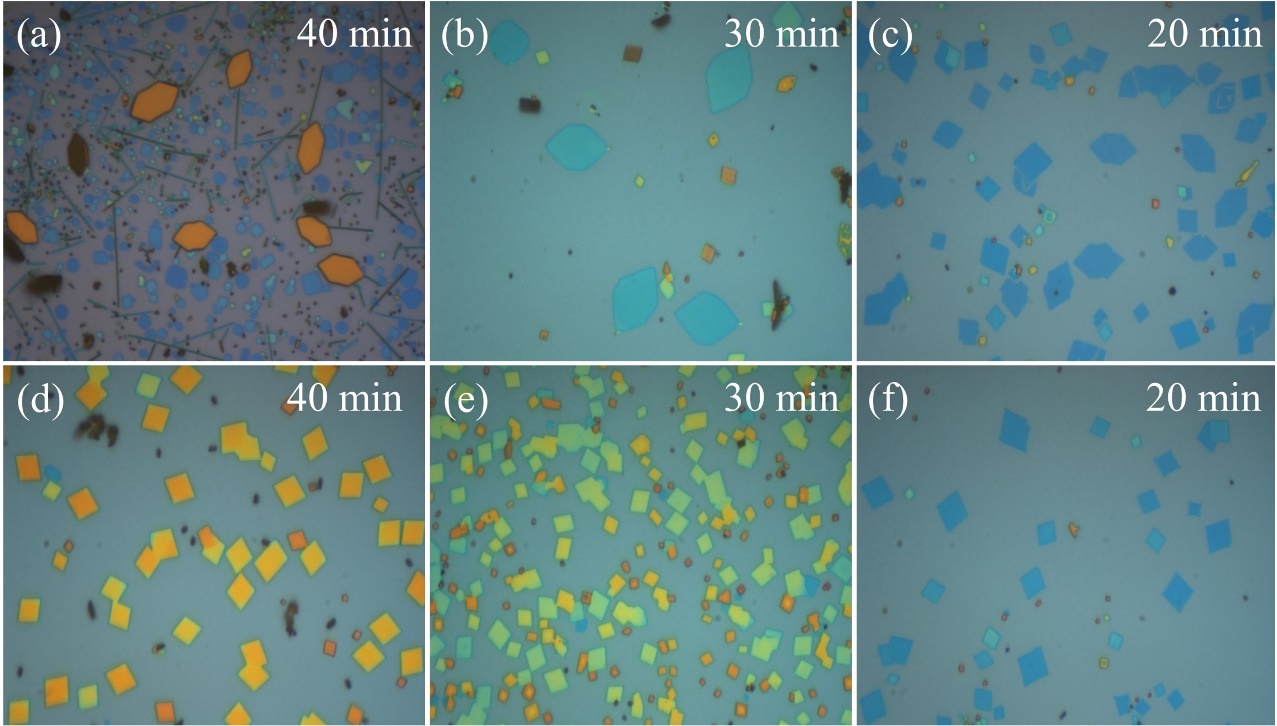


**Fig. S1.** The optical images reveal difference between thickness associated with different colors for both types of hexagonal and rectangular nanosheets. **(a-c)** The optical image with various thickness of hexagonal molybdenum oxides nanosheets with holding time **(a)** 40 min, (**b)** 30 min and **(c)** 20 min. **(d-f)** the optical images of rectangular molybdenum oxides nanosheets with holding time **(d)** 40 min, **(e)** 30 min and **(f)** 20 min

Figure S1 the large-scale image demonstrates that many identical nanosheets can be obtained by the CVD methods. According to our personal experience, the color and thickness depends on synthesis parameters i.e., holding temperature time, temperature variations, ramp rate of temperatures and distance between precursor and substrates.


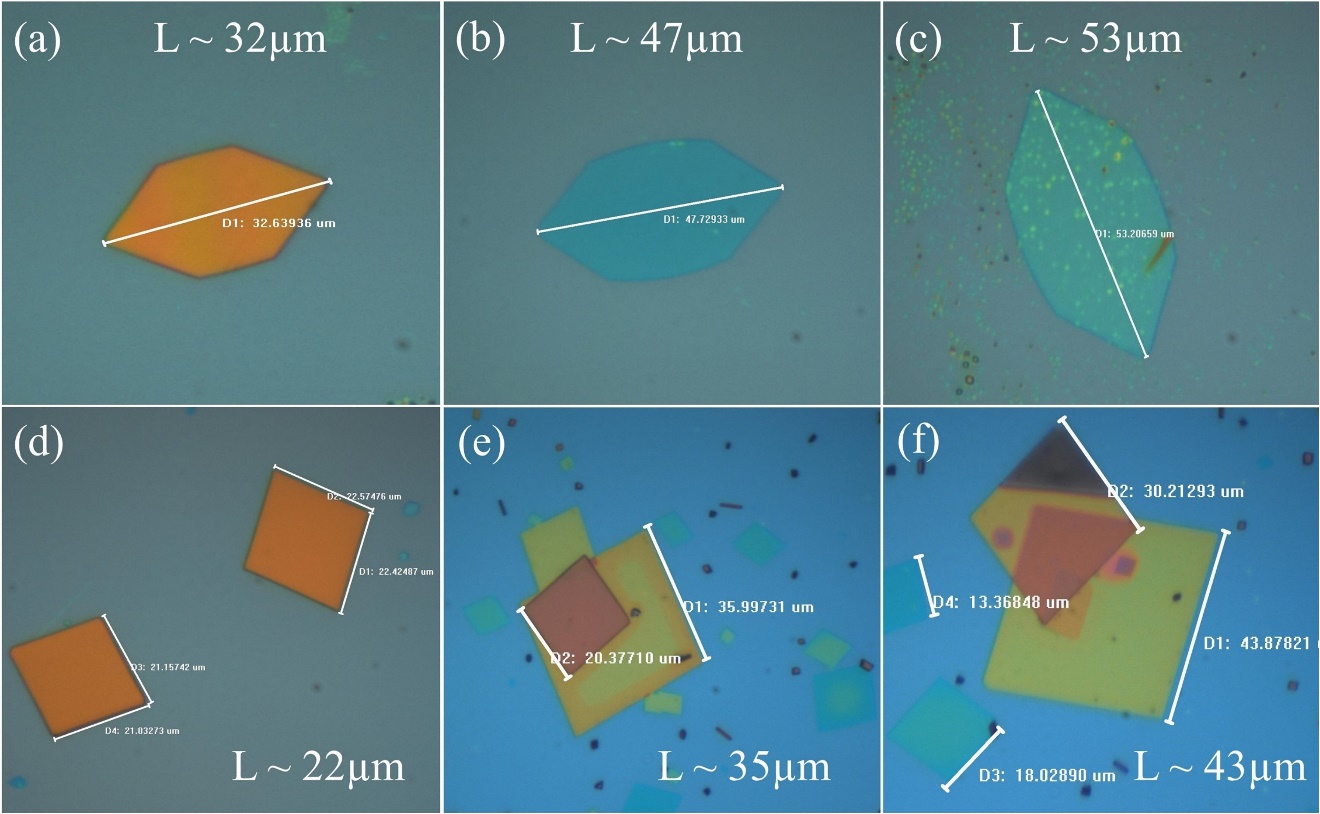


**Fig. S2.** **(a-c)** Optical microscopic images having different size and color of hexagonal molybdenum oxides nanosheets. **(d-f)** optical microscopic images having different size and color of rectangular molybdenum oxides nanosheets


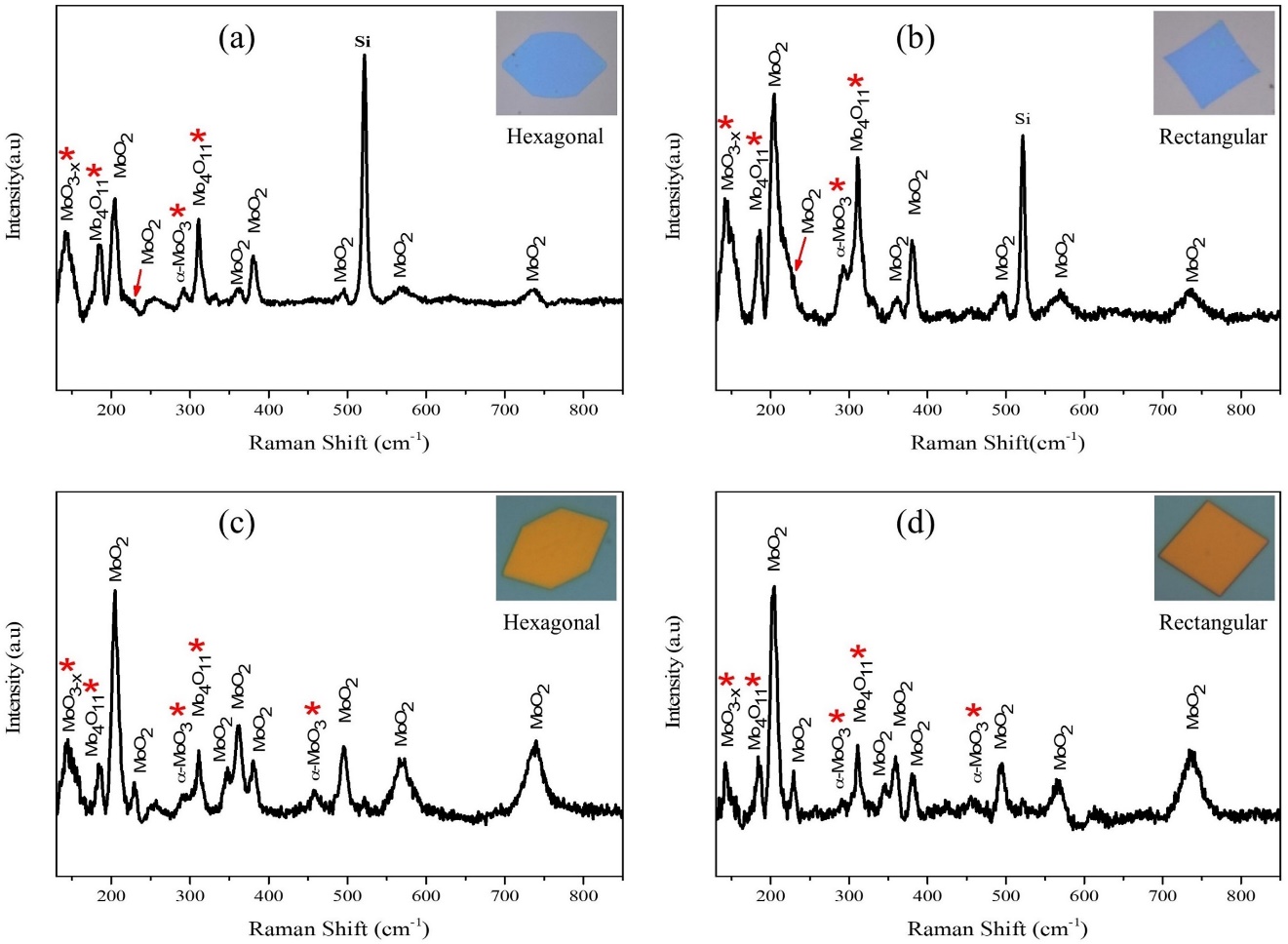


**Fig. S3.** Raman spectra of individual hexagonal and rectangular molybdenum dioxides nanosheets carried out through 532 nm laser (red-star shows other oxides rather than MoO_2_ i.e., MoO_3-x_, Mo_4_O_11_, α-MoO_3_). **(a)** Raman spectra of blue color hexagonal (thin) nanosheet; **(b)** Raman spectra blue color rectangular (thin) nanosheet. **(c)** Raman spectra of orange color hexagonal (thick) nanosheet. **(d)** Raman spectra orange color rectangular (thick) nanosheet


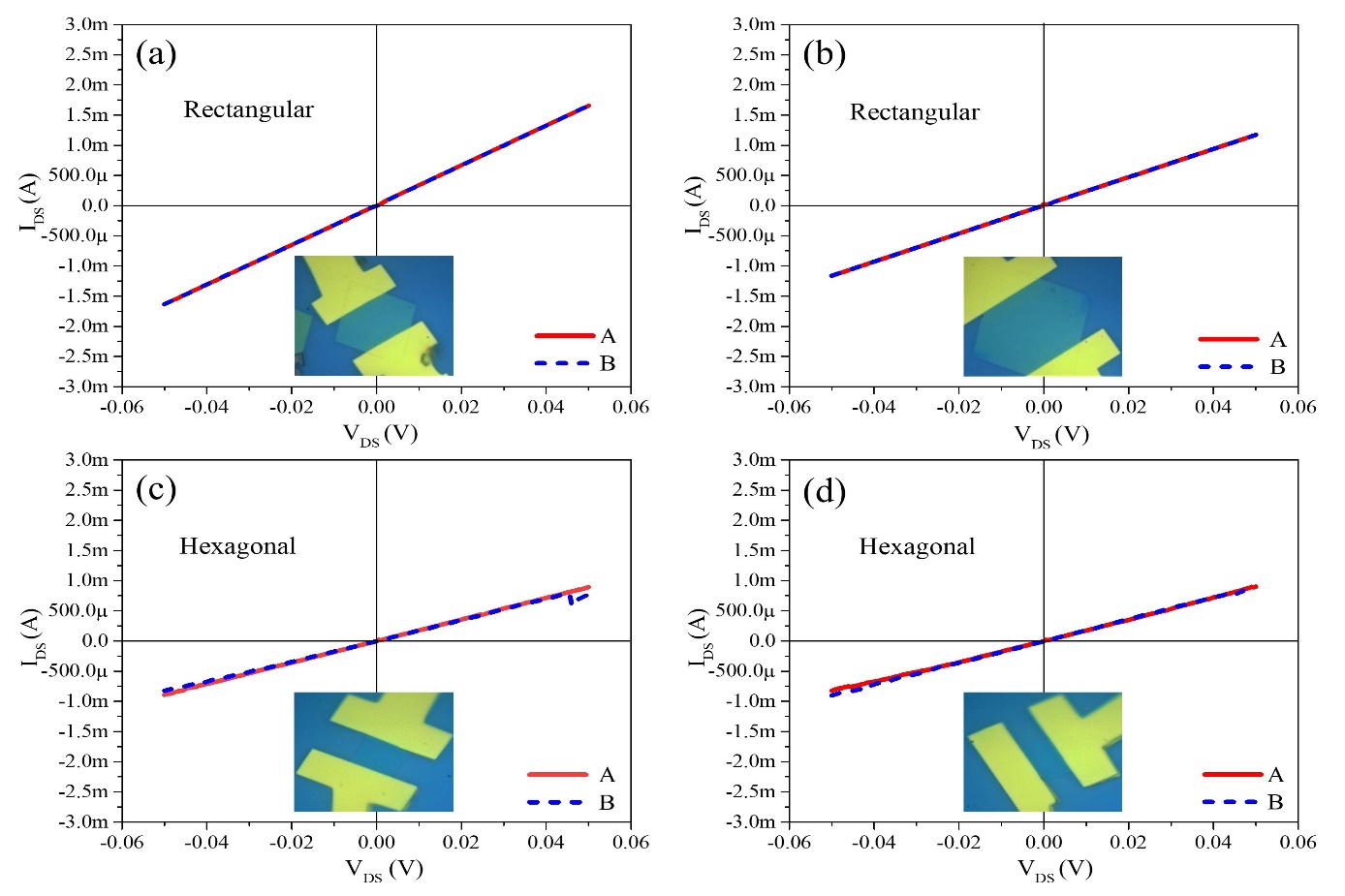


**Fig. S4.** The I-V curves of both; blue color thin rectangular and hexagonal nanosheets are measured by bias voltage (± 0.05 V). Furthermore, Ohmic equation utilized for resistance measurement. **(a)** I-V characteristics of individual single rectangular molybdenum oxide nanosheet’s device. Inset: Real image of the device. The resistance (R) is ~ 30 Ω at ± 0.05 V. **(b)** I-V characteristics of individual single rectangular molybdenum oxide nanosheet’s device. Inset: Real image of the device. The resistance (R) is ~ 43 Ω at ± 0.05 V. **(c)** I-V characteristics of individual single hexagonal molybdenum oxide nanosheet’s device. Inset: Real image of the device. The resistance (R) is ~ 61 Ω at ± 0.05 V. **(d)** I-V characteristics of individual single hexagonal molybdenum oxide nanosheet’s device. Inset: Real image of the device. The resistance (R) is ~ 61 Ω at ± 0.05 V.
